# Factors Determining Patients’ Choice Between Mobile Health and Telemedicine: Predictive Analytics Assessment

**DOI:** 10.2196/13772

**Published:** 2019-06-08

**Authors:** Saif Khairat, Songzi Liu, Tanzila Zaman, Barbara Edson, Robert Gianforcaro

**Affiliations:** 1 Carolina Health Informatics Program University of North Carolina at Chapel Hill Chapel Hill, NC United States; 2 School of Nursing University of North Carolina at Chapel Hill Chapel Hill, NC United States; 3 School of Information and Library Science University of North Carolina at Chapel Hill Chapel Hill, NC United States; 4 UNC Health Care Chapel Hill, NC United States

**Keywords:** mHealth, telemedicine, urgent care, predictive analytics

## Abstract

**Background:**

The solution to the growing problem of rural residents lacking health care access may be found in the use of telemedicine and mobile health (mHealth). Using mHealth or telemedicine allows patients from rural or remote areas to have better access to health care.

**Objective:**

The objective of this study was to understand factors influencing the choice of communication medium for receiving care, through the analysis of mHealth versus telemedicine encounters with a virtual urgent clinic.

**Methods:**

We conducted a postdeployment evaluation of a new virtual health care service, Virtual Urgent Clinic, which uses mHealth and telemedicine modalities to provide patient care. We used a multinomial logistic model to test the significance and predictive power of a set of features in determining patients’ preferred method of telecare encounters—a nominal outcome variable of two levels (mHealth and telemedicine).

**Results:**

Postdeployment, 1403 encounters were recorded, of which 1228 (87.53%) were completed with mHealth and 175 (12.47%) were telemedicine encounters. Patients’ sex (*P*=.004) and setting (*P*<.001) were the most predictive determinants of their preferred method of telecare delivery, with significantly small P values of less than .01. Pearson chi-square test returned a strong indication of dependency between chief concern and encounter mediums, with an extremely small *P*<.001. Of the 169 mHealth patients who responded to the survey, 154 (91.1%) were satisfied by their encounter, compared with 31 of 35 (89%) telemedicine patients.

**Conclusions:**

We studied factors influencing patients’ choice of communication medium, either mHealth or telemedicine, for a virtual care clinic. Sex and geographic location, as well as their chief concern, were strong predictors of patients’ choice of communication medium for their urgent care needs. This study suggests providing the option of mHealth or telemedicine to patients, and suggesting which medium would be a better fit for the patient based on their characteristics.

## Introduction

### Background

In the United States, approximately 19.3% of the population live in rural areas. With only 9% of the nation’s physicians practicing in such communities, the lack of health care providers in rural areas tends to be an intractable problem [1,2], causing rural residents to have a significantly lower health status than urban residents [3,4]. Aside from a shortage of health care staff, barriers to care due to the isolated location of residents and the lack of technology result in a poor quality of health care among rural populations [5,6]. Rural residents tend to use health care less due to the remoteness of where they live. For instance, colon cancer rates are high among rural residents, suggesting that they are less likely to receive timely cancer screening tests [7-10]. Rural populations are also at higher risk not only of cancer, but also of coronary heart disease [11]. Patients living close to a clinic tend to visit a health care provider more often than do patients living in rural areas [12]. Failure to obtain care on time may lead to a poor prognosis. These barriers to care for rural residents correlate with Hart’s inverse care law, which states that underserved populations have the worst access to health care [1,13]. Therefore, it is imperative to provide care to underserved populations.

The solution to the growing problem of rural residents lacking health care access may be found in the use of telemedicine and mobile health (mHealth). In telemedicine, the doctor-patient interaction is conducted by live video consultation [14,15]. Telemedicine not only improves health care accessibility for patients living in rural areas, but it is also expected to save US $4.3 billion annually [16,17]. Another method of providing care is through mHealth. mHealth is the use of mobile devices such as mobile phones, patient monitoring devices, personal digital assistants, and other wireless devices to provide medical care [18,19]. These two methods of providing care to patients remotely save significant travel costs for patients and their families, ensure that patients are seen in a timely manner, and help in-person care clinics or hospitals by reducing patient load [20].

Telemedicine is used in rural areas to educate patients, deliver teaching programs, and facilitate administrative meetings [21]. These help to reduce costs and save time. Use of teleoncology clinics in rural Kansas showed a cost reduction by almost 50%, from US $812 per consultation in 1995 to US $410 per consultation in 2000 [22]. Telemedicine can also be used to save time. The use of teleconsultation for veterans (individuals who previously served in the military) living a distance of 145 miles (233 km) from a health care facility was shown to save travel time of up to 142 minutes [23,24]. Apart from cost and time savings, telemedicine can be used to overcome barriers to health care access where conventional medical strategies do not apply [25]. Video consultation is very useful in providing consultation to patients in rural areas that lack a specialized physician. The Medical College of Georgia developed a Web-based telestroke system that enabled emergency physicians in rural areas to speak with specialists for patients with an episode of ischemic stroke. This system allowed physicians to examine patients using live video and to review medical imaging, and it recommended stroke therapies. Mean onset of stroke-to-treatment time was reduced by 20.2 minutes using the telestroke system, and only 2% of patients had a symptomatic hemorrhage [26]. Thus, patient outcomes were improved in an emergency situation. Lack of expert physicians in rural areas can create barriers for patients receiving urgent care [27]; therefore, video consultation can be effective in providing care to patients in critical conditions.

mHealth is an innovative way to deliver care. mHealth is used for remote monitoring and treating chronic diseases, to raise awareness, and for behavioral modification [28-34]. In one study, health data including blood pressure, pulse, weight, and dose of medication of patients with chronic heart failure and hypertension were transferred via a mobile phone, with an average data transfer accuracy of 83% (SD 22) [35]. This allowed physicians to remotely collect data for developing assessment and care management. Another study found that participants with controlled background displays on their mobile phones were likely to engage in a daily walk and cardiovascular exercise for 3 months, who otherwise would not have exercised [36]. Lastly, phone consultation was found to improve physical activities among women of low socioeconomic status who had high mortality rates due to high-risk behaviors [37].

### Objective

These two modalities, telemedicine and mHealth, improve access to care: telemedicine enables physician intervention, and mHealth promotes patients’ participation [38]. Yet less infrastructure being required for mHealth than for telemedicine, the rising popularity of mobile phones, the sophisticated third-generation network, and emerging ways to exchange information through mobile phones predict mHealth to be more promising for developing countries [39-41]. Although studies have shown the effectiveness of receiving care using mHealth and telemedicine, to the best of our knowledge, no study has compared patients’ preference for phone calls versus video conferencing based on their demographics, chief concern, and time spent in consultation. The objective of this study was to understand factors influencing patients’ choice of communication medium for receiving care, either through mHealth or in telemedicine encounters, when they were provided with both options in a virtual urgent clinic.

## Methods

### Study Design

We conducted a postdeployment evaluation of a new virtual health care service, Virtual Urgent Clinic (VUC), which uses mHealth and telemedicine modalities to provide patient care. VUC is a 24-hour-a-day, 7-day-a-week, on-demand service aimed at helping individuals with urgent medical needs to consult with a physician regarding their medical condition. The service was primarily designed to offer services regardless of the time of day or location of the patient in a more convenient form than the traditional in-person urgent care clinics. We obtained institutional review board approval from the University of North Carolina at Chapel Hill to conduct this research.

### Study Setting and Participants

VUC is cloud-based platform offered through a public website. Individuals with urgent medical needs can use VUC, despite their location, as long as they have access to a phone or a computer equipped with a microphone and camera with internet connection. Inclusion criteria for this study were individuals with a medical need who were over the age of 2 years. Exclusion criteria were patients under the age of 2 years, patients with no access to a phone or a computer with microphone and camera with internet connection.

### Materials

Individuals were required to create an account through the VUC website prior to scheduling a consultation. During the registration process, each individual had to fill out a short form providing basic demographic information. A secure link was sent to the individual’s email address for activation of the account. Once the account was activated, the individual indicated whom the e-visit was for and the intended provider type (eg, family physician). The website provided information regarding conditions not treatable through VUC, medications that VUC physicians could not prescribe, and important information regarding children under the age of 3 years. Once the individual verified having read this information, they were asked to fill out a series of short forms on the reason for the visit, their medical history, choice of pharmacy, choice of provider, payment, and confirmation. The cost of a VUC visit was a flat fee of US $49.

After the encounter, patients were asked to voluntarily participate in a short patient satisfaction survey. The survey aimed to solicit patients’ assessment of the encounter based on 4 criteria: (1) overall experience, (2) physician rating, (3) if they gave a fair or poor rating of the overall experience, their reason for the rating, and (4) open-ended patient comments.

### Outcomes

The primary outcomes were two predictive models that projected the users’ medium of choice given their demographics and chief concern. Secondary outcomes were encounter duration and satisfaction levels per encounter medium.

The dependent variable was encounter medium (mHealth, telemedicine). Independent variables were sex (female, male), age range (<18, 19-34, 35-49, ≥50 years), setting (urban, rural), insurance status (insured, uninsured), encounter time range (6 AM-12 PM, 12 PM-5 PM, 5 PM-12 AM, 12 AM-6 PM), day of the-week (weekday, weekend), top 20 chief concerns (Multimedia Appendix 1 shows a full list).

We included the top 20 chief concerns, which made up 68.57% (962/1403) of the total encounters, as a predictor instead of including all 148 concerns; we classified the remaining 128 encounters as others. The rationale behind this is that an excessive number of levels with a small number of data points would have added unnecessary complexity to the models.

### Statistical Analysis

We used multinomial logistic regression to build and compare the two models based on the predictive power of two sets of features in determining patients’ preferred method of telecare (mHealth and telemedicine) encounters. We selected the first set of independent variables to represent the demographics and socioeconomic status of the patient population. The additional feature, chief concern, captures patients’ self-reported reason for the telecare visit.

For model selection purposes, we used the step function in R version 3.6.0 (R Foundation) to eliminate the least significant predictors. The process started with the full model, where all predictors were included; it ceased when the current model reached its maximum performance measured by the Akaike information criterion (AIC) [42].

To measure the features’ predictive performance, we inferred the odds ratio (OR) by exponentiating the models’ coefficients. However, due to the lack of a simple and intuitive explanation of OR outcomes, we decided to follow previous research by interpreting OR as the risk ratio—the relative probability of an event happening in one group compared with another group [43]. We discuss this method’s limitations further below.

To evaluate the models’ prediction accuracy, we performed cross-validation with 70% of the original dataset training data and using 30% as the testing set. In addition, we measured the models’ efficiency and effectiveness using two common performance metrics: AIC and the simulated McFadden pseudo- *R*^2^.

We used several R packages for advanced analysis and model building: nnet for modelling the multinomial logistic regression function; mfx for calculating the relative risk ratio; and stargazer for rendering the summary statistics. We generated visualizations using Tableau version 9.0 (Tableau Software).

## Results

### Demographics

Postdeployment, 1403 encounters were recorded, of which 87.53% (1228) were completed with mHealth, and 175 (12.5%) were telemedicine encounters (Table 1). We tested two predictive models: one with a set of 6 demographic features extracted from the patients’ records and one with the chief concern feature as the predictor. We measured the results as the OR, indicating the magnitude of a specific feature’s predictive power. In addition, we analyzed the relationship between chief concern and the two significant demographic predictors—sex and setting. Subsequently, we evaluated and compared the difference between mHealth and telemedicine encounters, specifically the duration of consultation session, chief concern, the patients’ preference for alternative care-seeking options.

**Table 1:** Demographics of Virtual Urgent Clinic users.

Characteristics		Type of encounter		
		mHealth, n (%)	Telemedicine, n (%)	Total, n (%)
Number of encounters		1228 (87.53)	175 (12.47)	1403 (100.00)
**Sex**				
	Male	269 (82.01)	59 (17.99)	328 (23.38)
	Female	959 (89.21)	116 (10.79)	1075 (76.62)
**Age range (years)**				
	2-18	115 (83.94)	22 (16.06)	137 (9.76)
	19-34	434 (87.85)	60 (12.15)	494 (35.22)
	35-49	465 (88.24)	62 (11.76)	527 (37.56)
	≥50	214 (87.35)	31 (12.65)	245 (17.46)
**Setting**				
	Rural	569 (92.22)	48 (7.78)	617 (44.04)
	Urban	657 (83.80)	127 (16.20)	784 (55.96)
**Insurance status**				
	Insured	556 (91.15)	54 (8.85)	610 (43.48)
	Uninsured	672 (84.74)	121 (15.26)	793 (56.52)

**Table 2:** Odds ratio and significance (*P* value) of the demographic predictors^a^.

Predictor	Odds ratio	*P* value
Sex: male	1.662	.004
Setting: urban	2.014	<.001
Insurance status: uninsured	1.42	.06
Constant	0.064	<.001

^a^Reference group: telemedicine.

### Multinomial Logistic Regression Models

#### Predictive Model I: Demographics Features

Among the 6 predictors, sex and setting were the most predictive determinants of patients’ preferred method of telecare delivery, with significantly small *P* values of less than .01. Insurance status was not significant (*P*<.10). With all else held constant, patients from urban areas had 1.014 times greater odds than users from rural regions of using telemedicine than of using mHealth. Similarly, male patients had 66.2% greater odds than female patients with identical features of using telemedicine than of using mHealth, as Table 2 shows.

#### Predictive Model II: Top 20 Chief Concerns

Among the 20 chief concerns, 6 were significant predictors of patients’ preferred medium of telecare encounter (Table 3). A total of 4 predictors resulted in ORs greater than the neutral level of 1—urinary tract infection (*P*<.001), ear pain (*P*=.06), sinus infection (*P*=.04), and vaginal discharge (*P*<.001)—suggesting a lower tendency of choosing telemedicine over mHealth. Based on the model, we expected an 89% decrease in the odds of using telemedicine if a patient had a urinary tract infection. However, vaginal discharge yielded an OR of 0, indicating that no user with vaginal discharge chose telemedicine in this case. In contrast, pink eye (*P=*.05) and rash (*P*=.01) showed ORs greater than 1, suggesting a greater probability of opting for telemedicine. Based on the model, patients with pink eye were expected to have 1.39 times greater odds of choosing a telemedicine encounter.

**Table 3:** Odds ratio and significance (*P* value) of the chief concern predictor^a^.

Predictor	Odds ratio	*P* value
Urinary tract infection	0.11	<.001
Ear pain	0.256	.06
Pink eye	2.39	.05
Rash	2.325	.01
Sinus infection	0.5	.04
Vaginal discharge	0	<.001
Constant	0.168	<.001

^a^Reference group: telemedicine.

**Table 4:** Evaluation metrics of multinomial logistic regression models.

Model	Akaike information criterion	McFadden *R*^2^	Cross-validation prediction accuracy, %
Model I: demographics	1027.153	0.035	86.22
Model II: chief concerns	1030.168	0.064	86.22

#### Model Evaluation

The AIC of both models performed similarly, indicating that the two models were of similar complexity [44]. However, model II: chief concerns showed a slightly higher value (1030.168) than model I: demographics (1027.153), ranking model II: chief concerns lower than model I: demographics.

*R*^2^ of model II (0.064) was almost twice that of model I (0.035; Table 4). A higher value of *R*^2^ shows that a higher proportion of the dependent variable is explained by model II: chief concerns than by model I: demographics.

The cross-validation yielded a prediction accuracy of 86.22% (363 instances were correctly predicted out of the 421 data points in the testing set) for both models.

### Chief Concerns Analysis

Pearson chi-square test returned a strong indication of dependency between chief concern and encounter mediums, with a close-to-zero *P*<.001. We further examined the relationship between chief concern and the two significant predictors—sex and setting—and found the same strong correlations between the variables.

We analyzed the top 10 chief concerns of the two encounter methods, the results of which confirmed the difference between mHealth and telemedicine users’ primary reasons for seeking virtual urgent care. We observed a few extreme cases: for instance, urinary tract infection the most common concern among the mHealth users (n=147, 12.0% of a total of 1228 mHealth encounters), was absent from the telemedicine users’ top 10 list (Table 5). Conversely, telemedicine users, but not mHealth users, frequently consulted about eye-related problems (pink eye and eye swollen).

**Table 5:** Top 10 chief concerns in mobile health (mHealth) encounters (n=1228).

Chief concerns	Encounter medium: mHealth, n (%)	Sex: female, n (%)	Setting: rural, n (%)
Urinary tract infection	147 (11.98)	147 (100.0)	62 (42.2)
Sinus infection	129 (10.51)	113 (87.6)	62 (48.1)
Sore throat	116 (9.45)	94 (81.0)	58 (50.0)
Cough	82 (6.68)	53 (65)	49 (60)
Ear pain	42 (3.42)	27 (64)	23 (55)
Rash	37 (3.02)	23 (62)	24 (65)
Fever	32 (2.61)	22 (69)	16 (50)
Nasal congestion	31 (2.53)	24 (77)	19 (61)
Cold	30 (2.44)	25 (83)	12 (40)
Animal or insect bite or scratch	28 (2.28)	18 (64)	9 (32)

### Encounter Duration

The average duration of telemedicine encounters was 5.46 minutes, which is 5.4 percentage points higher than the mean duration of mHealth encounters (5.18 minutes). A Welch 2-sample *t* test refuted the null hypothesis of equal mean (*P*=.28, 95% CI –0.79 to 0.24) between the two samples, indicating the mean encounter durations of the two populations were significantly different.

**Figure 1:**
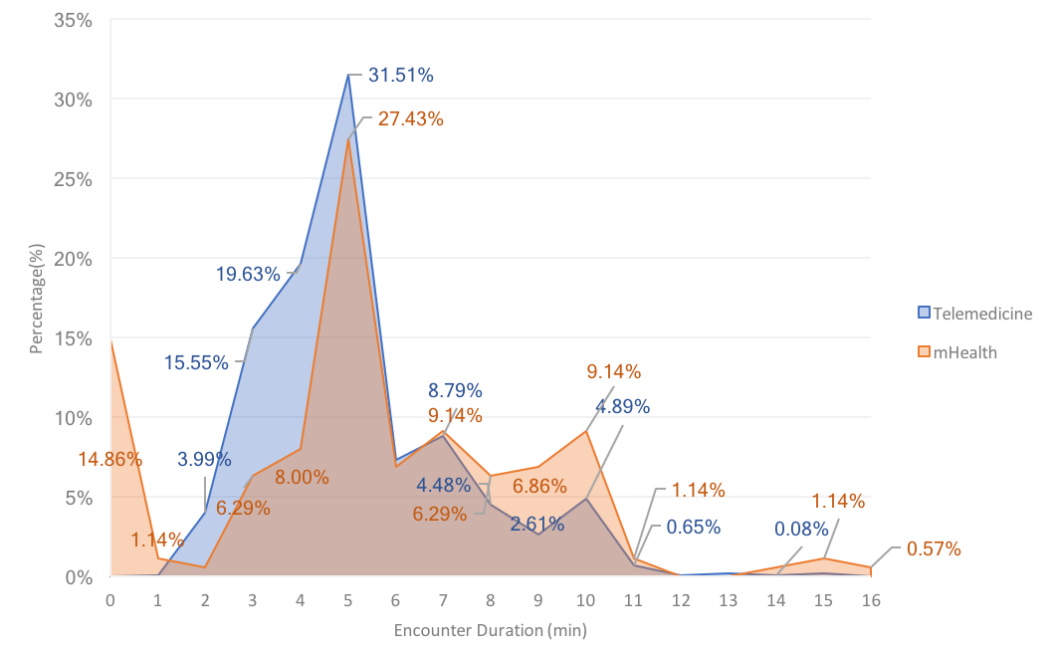
Distribution of encounter durations by encounter methods.

mHealth encounter duration had a range of 1 to 15 minutes, where 70.93% (871/1228) of the total encounters fell within the 1- to 5-minute range. Telemedicine encounters had a similar range of 0 to 16 minutes. Encounters lasting longer than 10 minutes accounted for 12.6% (22/175) of all telemedicine calls, double the 6.03% (74/1228) of mHealth encounters. In addition, 14.9% (26/175) of telemedicine calls lasted less than 1 minute, in contrast to the absence of mHealth calls of this length, as shown Figure 1 shows.

**Figure 2:**
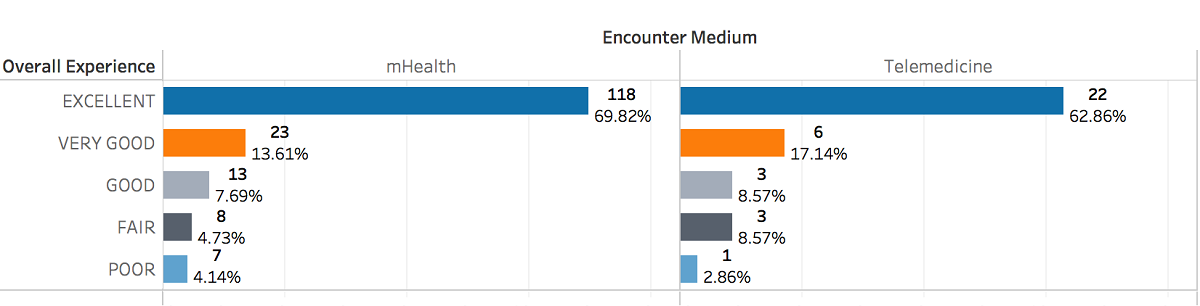
Self-reported overall experience satisfaction ratings. mHealth: mobile health.

#### Patient Satisfaction by Encounter Medium

For participants in all 1403 encounters, 204 (14.54%) responded to the satisfaction survey. High satisfaction levels were reported among both the mHealth and telemedicine groups. Of mHealth patients, 91.1% (154/169) were satisfied by their encounter compared with 89% (31/35) of telemedicine patients. A higher proportion of telemedicine patients (4/35, 11.4%) than mHealth patients (15/169, 8.9%) rated their experience as fair or poor (Figure 2).

#### Alternative Care-Seeking Options

**Figure 3:**
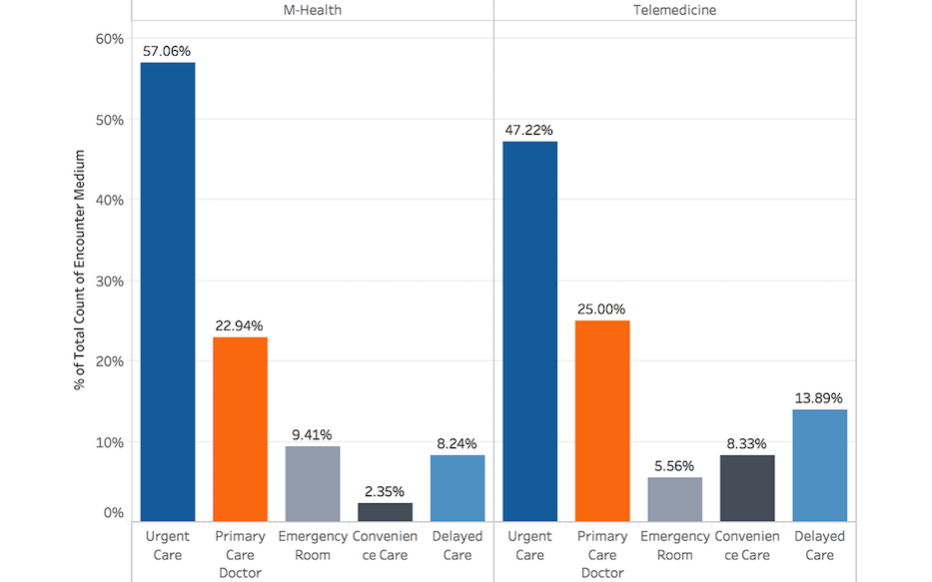
Alternative care-seeking choices of mobile health (mHealth) and telemedicine users.

We looked further into the telemedicine and mHealth users’ self-reported preferences for alternative care-seeking options. Patients were asked after their VUC consultation “if VUC was not available, which medical service would you have used?” The analysis revealed an almost identical distribution of users among the 5 options (Figure 3). In-person urgent care was the most popular alternative care option for both types of users. In addition, approximately one-fifth of the users would have delayed seeking care.

## Discussion

### Principal Findings

To our knowledge, this is the first study to comprehensively assess the effectiveness of providing patients with medium choice (phone call vs video call) of either mHealth or telemedicine to consult with physicians for urgent care needs. We leveraged a data science approach, namely, data analytics, to predict what factors informed patients’ choice of an mHealth or telemedicine medium. We analyzed the top 20 chief concerns in both groups to gain insight into the potential association between concern and choice of medium. Then, we analyzed the duration of encounters, self-reported alternative care-seeking options, and users’ responses to satisfaction surveys between both groups.

We proposed a model to predict the preferred choice of care delivery for patients. Patients’ sex and geographic location (rural or urban) significantly predicted their choice of care between mHealth and telemedicine. Patients from an urban area were twice as likely as users from rural regions to choose telemedicine over mHealth. Similarly, male patients were 1.6 times more likely than female patients with identical features to use telemedicine than mHealth. We conclude that male users from urban regions are the most likely to choose telemedicine over mHealth.

Patients’ chief concern significantly correlated with their choice of medium, where chief concern strongly correlated with mHealth or telemedicine. The duration of encounters was similar between both mediums, around the 5-minute mark. Overall, telemedicine encounters had a notable difference in range, from less than 1 minute up to 16 minutes. A possible justification for telemedicine encounters to last less than 1 minute needs to be studied in the future.

We observed that patients were satisfied with their choice of medium, as well as the service provided, which suggests that providers should offer the option of mHealth or telemedicine to their patients and allow them to choose. We recommend considering patients’ sex and setting as predictive factors to provide suggestions on which communication medium would best fit patients based on their characteristics. Patient satisfaction was high in both groups, with higher dissatisfaction among telemedicine users, which may be attributed to the quality of the video or audio feed. There was no significant difference between the groups in terms of their self-reported responses to alternative care-seeking options.

### Strengths and Limitations

A strength of this research is the ability to alleviate the demand on in-person urgent care clinics and emergency rooms by providing a virtual clinic where patients can be seen and treated. Since VUC is an on-demand and cloud-based service, there was no purposive sampling, which allows the findings of this study to be more generalizable. The digital nature of the service may introduce bias to the sample population; however, this study focused on two digital interventions and, therefore, if any bias was introduced, it should not have influenced the study findings. Another strength is the convenience of providing both mHealth and telemedicine options to patients within the same platform without further setup. The response rate of the voluntary survey was adequate given that we provided no incentive to participate.

One limitation of this study is the lower number of telemedicine encounters relative to mHealth encounters, which can be attributed to several factors, such as personal preference, time of the call, access to a Web camera, and internet connection speed. Another limitation is the absence of information regarding the reason for telemedicine encounters ending in less than 1 minute. This study can be further strengthened by capturing patient outcomes after the consultation visit by looking at 30-day hospitalization rates to assess the quality of care for each medium, which is a future direction of this research.

### Conclusion

We studied factors influencing patients’ choice of communication medium, either mHealth or telemedicine, for a virtual care clinic. Patients’ preference for mHealth or telemedicine was significantly influenced by their sex and geographic location, as well as their chief concern. Despite other preferences, patients were highly satisfied by their choice of communication medium. This study showed that providing the option of mHealth or telemedicine to patients suggests which medium would be a better fit for patients based on their characteristics.

## Appendix

Multimedia Appendix 1 Top 20 chief concerns.

## Abbreviations

AIC: Akaike information criterion
mHealth: mobile health
OR: odds ratio
VUC: Virtual Urgent Clinic
