# Supplementary material for: Factors Determining Patients’ Choice Between Mobile Health and Telemedicine: Predictive Analytics Assessment
Source: JMIR Mhealth Uhealth. 2019 Jun 8;7(6):e13772. doi: 10.2196/13772 (PMC6592402; doi:10.2196/13772)
Supplement: Multimedia Appendix 1 [file mhealth_v7i6e13772_app1.pdf]

Multimedia Appendix 1: Top 20 chief concerns.

Chief complaints	Counts
Urinary tract infection	150
Sinus infection	141
Sore throat	136
Cough	92
Rash	53
Ear pain	44
Fever	41
Nasal congestion	35
Animal or insect bite or scratch	34
Cold	32
Headache	29
Refill	28
Pink eye	26
Yeast infection	23
Allergy	18
Congestion	18
Dental	18
Urination problem	16
Vaginal discharge	15
Ear congestion	13
Others	441
